# Cutting-Edge Cardiology: Individualizing Antiplatelet Therapy for Optimal Acute Coronary Syndrome Outcomes

**DOI:** 10.7759/cureus.112849

**Published:** 2026-07-17

**Authors:** Gilbert Abounader, Sarah Bejjani, Said M Chaaban, Sobhi Dada, Robert Fakhoury, Malek Hamade, Robert Hobeika, Mohamad Iskandarani, Malek Mohammed, Abdallah G Rebeiz, Ali El Sayed, Marwa Hallal, Maroun Soueidy

**Affiliations:** 1 Department of Cardiology, Lebanese Hospital Geitawi, Beirut, LBN; 2 Department of Medical Affairs, Algorithm SAL, Beirut, LBN; 3 Department of Interventional Cardiology, Beirut Arab University, Beirut, LBN; 4 Cardiology Division, Hammoud Hospital University Medical Center, Saida, LBN; 5 Department of Cardiology, Lebanese American University School of Medicine, Beirut, LBN; 6 Department of Interventional Cardiology, Beirut Cardiac Institute Rassoul Aazam Hospital, Beirut, LBN; 7 Department of Interventional Cardiology, Lebanese Hospital Geitawi, Beirut, LBN; 8 Department of Cardiovascular Disease, Bahman Hospital, Beirut, LBN; 9 Department of Cardiology, American University of Beirut Medical Center, Beirut, LBN; 10 Department of Interventional Cardiology, Al-Zahraa Hospital University Medical Center, Beirut, LBN; 11 Department of Dermatology, Gilbert and Rose-Marie Chagoury School of Medicine, Lebanese American University, Byblos, LBN; 12 Department of Interventional Cardiology, Mount Lebanon Hospital, Beirut, LBN; 13 Department of Interventional Cardiology, Sacre Coeur Hospital, Beirut, LBN

**Keywords:** acute coronary syndromes, coronary artery disease, dual antiplatelet therapy, individualized treatment, percutaneous coronary intervention

## Abstract

Dual antiplatelet therapy (DAPT), consisting of aspirin and a P2Y12 inhibitor, is fundamental after percutaneous coronary intervention (PCI) in acute coronary syndromes (ACSs). Recent evidence supports moving from a uniform to an individualized approach, tailoring therapy duration and intensity to patient-specific ischemic and bleeding risks. A targeted literature review was conducted in PubMed, MEDLINE, Scopus, and Google Scholar from inception to June 2025. Relevant MeSH and free-text terms included "acute coronary syndromes, dual antiplatelet therapy, percutaneous coronary intervention, ticagrelor, prasugrel, clopidogrel, de-escalation, abbreviated therapy, rivaroxaban, and dual pathway inhibition." Boolean operators were applied, and studies were restricted to English-language publications in humans. Case reports, small case series, and studies unrelated to ACS populations were excluded. In parallel, a panel of 10 interventional cardiologists from major Lebanese institutions convened in Beirut in 2025. Experts critically appraised global trial data and contextualized recommendations within regional realities of patient risk profiles, drug accessibility, and healthcare infrastructure. Guidelines and pivotal trials endorse tailoring DAPT duration: shortened courses (1-3 months) with P2Y12 monotherapy in high-bleeding-risk patients, extended therapy beyond 12 months or dual-pathway inhibition in high-ischemic-risk patients, and preferential use of ticagrelor or prasugrel over clopidogrel in ACS. Expert consensus emphasized balancing bleeding and ischemic risks, incorporating validated risk scores, and adapting strategies to socioeconomic constraints in Lebanon and the wider MENA (Middle East and North Africa) region. By integrating global evidence with regional expert insights, this review provides a pragmatic framework for precision-based DAPT strategies in ACS, optimizing outcomes while minimizing harm.

## Introduction and background

Dual antiplatelet therapy (DAPT), typically aspirin plus a P2Y12 inhibitor, is a cornerstone after percutaneous coronary intervention (PCI) and acute coronary syndromes (ACSs) to prevent stent thrombosis and recurrent ischemic events [1]. Aspirin and P2Y12 inhibitors target complementary pathways of platelet activation, providing more effective inhibition of thrombus formation than either agent alone during the high-risk period following PCI [1]. However, the optimal DAPT strategy balances thrombotic protection against bleeding risk, and recent evidence has shifted practice from a “one-size-fits-all” approach to a more individualized regimen [2,3]. Over the past few years, major clinical trials and updated guidelines from cardiology societies have refined recommendations on DAPT duration, intensity, and agent selection. These guidelines emphasize tailoring therapy duration to patient risk profiles [4]. 

Regional epidemiology and risk stratification insights

Coronary heart disease (CHD) is on the rise across the Middle East and North Africa (MENA), now ranking as the region’s leading cause of death. Patients often present with ACS at younger ages, carrying a heavier burden of risk factors. In 2019, the age-standardized prevalence of ischemic heart disease in MENA reached 5,353 per 100,000, with mortality at 174.4 per 100,000 [5].

In Lebanon, CHD represents a substantial and growing public health burden. National data demonstrate a high prevalence of CHD among adults aged ≥40 years, particularly in men and older individuals [6]. In addition, urban cohorts reveal a high prevalence of modifiable cardiovascular risk factors, including obesity, hypertension, dyslipidemia, diabetes, and cigarette smoking [7]. These findings highlight the need for optimized cardiovascular risk management and individualized antiplatelet strategies. Given the

unique regional patient characteristics, healthcare infrastructure, and variations in treatment accessibility, practical recommendations informed by local expert experience are needed to complement international guideline recommendations.

**Figure 1:**
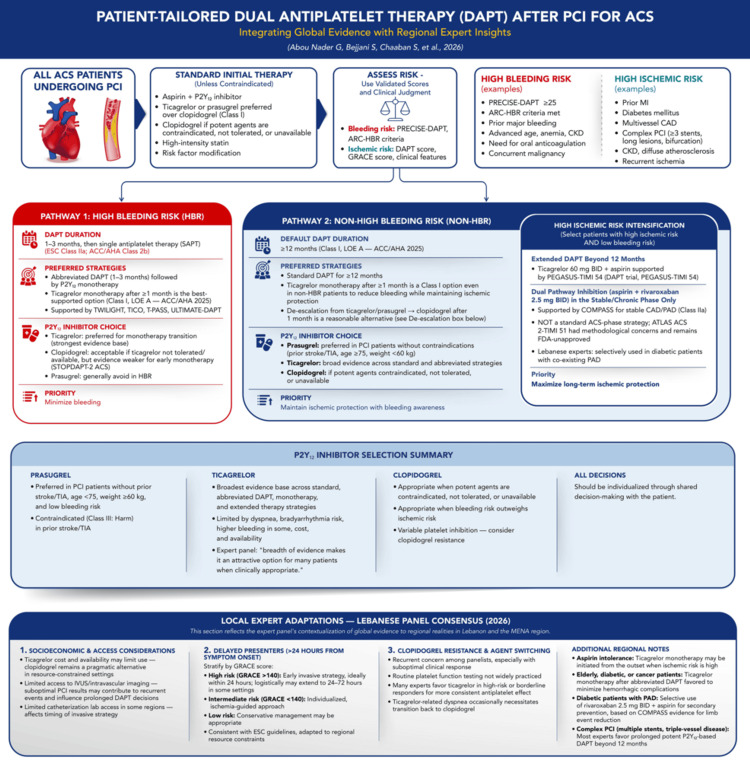
Patient-Tailored Dual Antiplatelet Therapy After PCI for Acute Coronary Syndromes: A Practical Clinical Framework PCI, percutaneous coronary intervention; DAPT, dual antiplatelet therapy; ACS, acute coronary syndrome. Figure credits: Author, using Adobe Illustrator (Adobe Inc., San Jose, CA, USA); generative AI has not been used to create this image.

Accordingly, this review synthesizes evidence from contemporary clinical trials and international guidelines with the perspectives of a Lebanese expert panel to provide a pragmatic framework for individualizing DAPT strategies in patients with ACS. By integrating global evidence with regional clinical experience, the manuscript aims to facilitate the implementation of patient-tailored DAPT recommendations in local healthcare settings. A summary of the clinical framework (Figure 1) distinguishes guideline-supported recommendations from regional expert adaptations to facilitate practical implementation in daily clinical practice.

## Review

Materials and methods

A targeted literature review was conducted using PubMed, MEDLINE, Scopus, and Google Scholar from database inception through June 2025. Relevant Medical Subject Headings (MeSH) and free-text terms included acute coronary syndromes, dual antiplatelet therapy, percutaneous coronary intervention, ticagrelor, prasugrel, clopidogrel, de-escalation, abbreviated therapy, rivaroxaban, and dual pathway inhibition. Boolean operators were applied to refine results. The search was restricted to studies published in English. Studies were excluded if they were not related to human subjects, addressed non-ACS cardiovascular populations without implications for DAPT, or consisted solely of case reports or small case series.

In parallel, a panel of 10 interventional cardiologists from leading Lebanese institutions was convened. Panelists were selected based on their recognized expertise in ACS management, academic contributions, and representation of diverse healthcare settings. Structured expert discussions were held in Beirut in 2025 to integrate current clinical evidence with regional experience and address local realities, including patient risk profiles, drug accessibility, and healthcare infrastructure. Where recommendations required agreement, consensus was established through structured discussion followed by voting among panel members.

Results

Current Guideline Recommendations on DAPT Duration and Intensification

Contemporary guidelines from both sides of the Atlantic have converged on a patient-tailored approach to DAPT after coronary stenting. The 2025 American College of Cardiology (ACC)/American Heart Association (AHA)/American College of Emergency Physicians (ACEP)/National Association of EMS Physicians (NAEMSP)/Society for Cardiovascular Angiography and Interventions (SCAI) Guidelines for the Management of Patients With ACS and the latest 2023 European Society of Cardiology (ESC) Guidelines for the management of ACS outline baseline DAPT durations with flexibility for earlier modification in select patients [4,8]. Key recommendations include the following:

Standard DAPT duration: The ESC (2017/2023) and ACC/AHA (2016) guidelines recommend 12 months of DAPT after ACS (Class I), though the 2023 ESC ACS guidelines now consider six months reasonable (Class IIa) for many patients [8,9]. For elective PCI (stable coronary artery disease (CAD)), both the ESC and ACC/AHA recommend six months of DAPT as the default (Class I), with the 2021 ACC/AHA/SCAI and 2023 AHA/ACC CCD guidelines further endorsing 1-3 months of DAPT followed by P2Y12 inhibitor monotherapy as a reasonable alternative (Class IIa). In patients with high bleeding risk, DAPT may be shortened to three months or even one month in both settings [4,8].

High-bleeding-risk patients: In high bleeding risk, 1-3 months of DAPT then P2Y12 monotherapy is supported (MASTER DAPT) [1,10].

High-ischemic-risk patients: For high ischemic risk, extended DAPT (>12 months) reduces events (DAPT, PEGASUS), though bleeding risk rises [11,12]. Notably, guidelines specify that the second antithrombotic agent in extended therapy can be either a P2Y12 inhibitor (such as ticagrelor 60 mg twice daily, per PEGASUS) or very-low-dose rivaroxaban 2.5 mg twice daily added to aspirin, both having shown outcome benefits in clinical trials [13,14].

Agent selection in ACS: Ticagrelor or prasugrel is preferred over clopidogrel in ACS (PLATO, TRITON), with clopidogrel reserved for contraindications or unavailability [1]. Clopidogrel remains recommended if the newer agents are not available or are contraindicated (for example, prasugrel is contraindicated in patients with prior stroke/transient ischemic attack (TIA), and ticagrelor may be avoided in severe asthma/COPD or profound bradycardia); in such cases, clopidogrel is an acceptable alternative, underscoring the need to tailor the regimen to each patient’s clinical scenario.

The default 6-12 months of DAPT may be shortened for bleeding risk or extended for ischemic risk [11,12]. Risk scores such as PRECISE-DAPT and DAPT aid decisions on shortening or prolonging therapy [1]. 

Ticagrelor vs. Clopidogrel in ACS: Efficacy and Safety Insights

The PLATO trial was pivotal in establishing ticagrelor’s superiority over clopidogrel in ACS. Ticagrelor significantly reduced the composite of cardiovascular death, myocardial infarction (MI), or stroke compared with clopidogrel, without a significant increase in overall major bleeding, although non-CABG-related major bleeding occurred more frequently with ticagrelor [15].

Similarly, prasugrel in the TRITON-TIMI 38 trial showed that prasugrel reduced ischemic events vs clopidogrel but increased bleeding; it is contraindicated after prior stroke/TIA and used cautiously in older or low-weight patients [1,4,8,16]. Meta-analyses suggest that ticagrelor’s ischemic benefit is modest outside trials, while bleeding risk is consistently higher; some pooled data still show reductions in mortality and MI [17-19].

DAPT Duration: Shortening and De-escalation Strategies

Optimizing the duration of DAPT after PCI has been a major focus of recent trials, especially as stent technology has improved and bleeding mitigation has become a priority. The traditional 12-month DAPT for everyone after ACS (or 6-12 months after elective PCI) is now tempered by a wealth of data on shorter regimens and on switching strategies. Two major approaches to safely abbreviate DAPT have emerged:

(1) Early aspirin withdrawal (with continuation of a single P2Y12 inhibitor), and (2) DAPT “de-escalation” (switching from a potent P2Y12 inhibitor to a less potent agent, typically ticagrelor/prasugrel to clopidogrel, after an initial period). Both aim to reduce bleeding while preserving antithrombotic efficacy, and both have gained traction in recent years [1].

For patients who are not at high bleeding risk but on potent DAPT for ACS, several trials support dropping aspirin after a few months and continuing P2Y12 inhibitor monotherapy. In the large TWILIGHT trial, high-risk post-PCI patients (including many ACS) who completed three months of DAPT were randomized to aspirin cessation vs. DAPT continuation; ticagrelor was continued in both arms. Aspirin-free therapy led to a 44% reduction in major bleeding with no increase in ischemic events in the overall cohort. Notably, in the ACS subgroup of TWILIGHT, ticagrelor monotherapy likewise showed significantly less bleeding than ticagrelor+aspirin, with no higher risk of death, MI, or stroke [20].

The TICO trial extended this concept exclusively in ACS patients (including ST-elevation myocardial infarction (STEMI)): a strategy of aspirin for only one month followed by ticagrelor alone for one year was superior to 12-month DAPT in reducing bleeding, without any penalty in ischemic outcomes [21]. This approach is guideline-endorsed. The 2025 AHA guidelines state that, in patients with ACS undergoing PCI who have tolerated DAPT with ticagrelor, transition to ticagrelor monotherapy after ≥1 month may be considered (Class IIa) [4].

A critical caveat is which P2Y12 inhibitor is used if aspirin is discontinued early. The trials demonstrating favorable outcomes (TWILIGHT and TICO) evaluated ticagrelor monotherapy following a short course of DAPT, leveraging its greater antiplatelet potency [20,21]. In contrast, the STOPDAPT-2 ACS trial evaluating clopidogrel monotherapy after one month of DAPT did not demonstrate an increase in ischemic events but failed to establish noninferiority for ischemic outcomes, despite reducing bleeding [22]. These findings suggest that the evidence supporting early P2Y12 inhibitor monotherapy after ACS is stronger for ticagrelor than for clopidogrel.

Clopidogrel’s variability and weaker effect make it suboptimal alone in the high-risk early post-ACS period [1]. Thus, if a patient’s regimen involves clopidogrel (for example, due to intolerance of ticagrelor/prasugrel), most experts would not shorten DAPT to <6-12 months in an ACS setting unless bleeding risk absolutely mandates it. Instead, alternative strategies like de-escalation could be used for such patients.

DAPT de-escalation refers to switching the P2Y12 inhibitor to a less potent option or reducing its dose after the acute phase, rather than stopping aspirin early. This strategy acknowledges that ischemic risk is highest in the initial period after ACS and stenting, while bleeding risk is cumulative and may become more problematic later on [1]. By stepping down the intensity of DAPT over time, one might achieve a better risk balance. Several trials have explored this:

The TOPIC trial randomized ACS patients who had completed one month of DAPT with aspirin + a potent P2Y12 inhibitor (ticagrelor/prasugrel) to either continue that or de-escalate to aspirin + clopidogrel for the remainder of the year. De-escalation led to a significant reduction in bleeding with no increase in ischemic events, suggesting it was a safer strategy in that cohort [23].

The more recent TALOS-AMI trial specifically examined unguided de-escalation from ticagrelor to clopidogrel after one month in patients with acute MI. This large trial (over 2,600 patients) actually found that de-escalation was not only noninferior but superior to continuing ticagrelor: the composite endpoint of cardiovascular death, MI, stroke, or Bleeding Academic Research Consortium (BARC) ≥2 bleeding at one year occurred in 4.6% of the de-escalation group vs. 8.2% with continued ticagrelor (HR 0.55, p<0.001). The benefit was driven mainly by less bleeding, without an uptick in ischemic events, affirming that switching to clopidogrel after the first month in stabilized MI patients can improve net outcomes. It is worth noting that this trial was in an East Asian population (possibly more clopidogrel-sensitive); still, it provides robust evidence for the safety of unguided de-escalation in ACS after the highest-risk early phase is past [24].

Other studies like TROPICAL-ACS used a guided approach (platelet function testing to decide de-escalation) and similarly showed de-escalation (prasugrel to clopidogrel after 1-2 weeks if platelet function was adequate) was noninferior to continued prasugrel, with bleeding reduction [25]. Additionally, the POPular Genetics trial (2020) employed genetic testing to guide P2Y12 inhibitor selection in STEMI (switching carriers of CYP2C19 loss-of-function to ticagrelor, others to clopidogrel) and found reduced bleeding and similar ischemic outcomes -- essentially a personalized de-escalation approach [26].

De-escalation of DAPT, such as switching from ticagrelor to clopidogrel, has emerged as a reasonable strategy in patients with ACS who develop side effects, bleeding concerns, or cost limitations. A 2020 pooled analysis of ~10,800 ACS patients showed that both guided and unguided de-escalation significantly reduced clinically relevant bleeding, with no safety signal when switching from ticagrelor [27,28]. Current guidelines now endorse de-escalation as an acceptable strategy: for example, the ESC 2020 guidance for ACS allows switching from a potent agent to clopidogrel after the acute phase in patients deemed to have high bleeding risk or other needs (Class IIb) [19].

Individualized clinical judgment remains central to optimizing DAPT duration following PCI. Given the heterogeneity of patient risk profiles, a uniform approach to DAPT is no longer appropriate. Instead, therapeutic decisions should be guided by a careful assessment of both bleeding and ischemic risks [1]. For patients identified as having a high bleeding risk, shortened DAPT followed by single antiplatelet therapy (SAPT), typically within one to three months, has emerged as a preferred strategy and is supported by the 2020 ESC guidelines as a Class IIa recommendation [19]. In contrast, de-escalation of DAPT intensity, such as transitioning from ticagrelor to clopidogrel, is recognized as a Class IIb recommendation by the ESC guidelines. The 2025 ACC/AHA guidelines recommend that, in patients with ACS who have tolerated DAPT with ticagrelor, transition to ticagrelor monotherapy ≥1 month post-PCI is useful to reduce bleeding risk (Class I, LOE A). This recommendation is supported by evidence from the T-PASS and ULTIMATE-DAPT trials, which demonstrated that abbreviated one-month DAPT followed by ticagrelor monotherapy reduced bleeding without compromising ischemic outcomes in appropriately selected patients [4,29,30].

Rivaroxaban 2.5 mg in ACS and CAD: Dual-Pathway Antithrombotic Therapy

Another innovative strategy in the antithrombotic arsenal is the addition of a very-low-dose anticoagulant (rivaroxaban 2.5 mg twice daily) on top of antiplatelet therapy -- often referred to as “dual pathway inhibition.” This regimen, which targets both platelet aggregation and thrombin generation, has gained strong expert and guideline support for use in stable atherosclerotic disease, based on the COMPASS trial, where it significantly reduced the risk of cardiovascular death, MI, or stroke by 24% compared to aspirin alone [31]. Although major bleeding was more frequent, most events were nonfatal and not intracranial, resulting in a favorable net clinical benefit. Based on COMPASS, major guidelines have incorporated rivaroxaban 2.5 mg BID + aspirin as a Class IIa recommendation in patients with stable CAD/peripheral artery disease (PAD) who are at high ischemic risk but without high bleeding risk, such as those with polyvascular disease, recurrent events, or prior MI with multiple high-risk features [31]. This indication is now approved in Europe and increasingly adopted in global practice. While rivaroxaban 2.5 mg BID was also evaluated in the ATLAS ACS 2-TIMI 51 trial in the post-ACS setting, the increased bleeding risk and methodological concerns have limited its uptake, and it remains unapproved by the FDA [32].

Discussion

Expert Perspectives on Individualized DAPT Strategies

During the panel discussion, interventional cardiologists engaged in a comprehensive exchange of insights, drawing on both clinical trial data and firsthand experience in real-world practice. Experts highlighted that ticagrelor monotherapy following an abbreviated DAPT course is gaining favor in patients at high risk of bleeding, particularly in light of findings from the TWILIGHT trial. In such cases, aspirin is typically discontinued after one month post-PCI, while ticagrelor 90 mg BID is continued as monotherapy for an extended period. This approach was viewed as especially beneficial in elderly patients, individuals with diabetes, or those with concurrent malignancies, where the risk of hemorrhagic complications is elevated. Experts emphasized that this strategy allows for a meaningful balance between minimizing bleeding risk and maintaining sufficient ischemic protection. In contrast, when treating patients with multiple stents, triple-vessel disease, or anatomically complex lesions, most experts favored continuation of potent P2Y₁₂ inhibitor-based therapy beyond the initial 12 months. The need for prolonged DAPT in such populations was justified by their heightened thrombotic risk. On the other hand, low-risk individuals, typically younger patients with single-vessel disease and no adverse procedural features, were seen as appropriate candidates for de-escalation to monotherapy after the first year. Across the panel, there was consensus that antiplatelet strategies should be tailored based on individual ischemic and bleeding profiles rather than fixed timelines.

P2Y12 Inhibitor Selection and Clopidogrel Resistance

Clopidogrel resistance was a recurrent concern raised by several experts, particularly in cases of suboptimal clinical response or recurrent thrombotic events. Although routine platelet function testing is not widely practiced, many panelists favored ticagrelor in high-risk or borderline responders, citing its more consistent and potent antiplatelet effect. Nevertheless, the use of ticagrelor remains occasionally limited by side effects such as dyspnea, which in some instances led to a transition back to clopidogrel. In general, clopidogrel was reserved for patients with lower ischemic risk or when ticagrelor was contraindicated, not tolerated, or inaccessible due to cost.

Regional Practice Challenges

Several experts underscored that recurrent ischemia and late stent thrombosis are often linked to mechanical factors such as geographic stent miss or underexpansion. These technical pitfalls, while well-recognized, remain under-addressed due to limited access to intravascular imaging modalities like intravascular ultrasound, which is not routinely available in many Lebanese institutions. As such, suboptimal PCI outcomes were acknowledged as an ongoing contributor to recurrent ACS presentations and one of the factors influencing prolonged DAPT decisions.

Ticagrelor monotherapy was also considered appropriate in real-world scenarios where patients are unable to tolerate aspirin, whether due to hypersensitivity or gastrointestinal complications. In these cases, experts reported initiating ticagrelor alone at the outset of therapy, particularly when the ischemic risk was deemed high enough to preclude the option of minimal antiplatelet coverage. This practice was in line with international guidelines that endorse P2Y₁₂ monotherapy in patients with clear contraindications to aspirin.

Though less frequently applied in routine practice, the use of rivaroxaban 2.5 mg BID in combination with aspirin for secondary prevention was acknowledged by a few experts. This regimen was selectively employed in diabetic patients with co-existing PAD, based on evidence suggesting a reduction in major limb events, including amputation. However, broader implementation was constrained by concerns regarding bleeding risk and challenges related to drug cost and reimbursement in the local setting.

During the expert discussion, Lebanese interventional cardiologists also emphasized the unique challenges in ACS management posed by limited access to catheterization labs, varying patient risk profiles, and socioeconomic barriers. The management of patients presenting >24 hours to 28 days after symptom onset sparked significant debate. While some favored urgent catheterization regardless of timing, others advocated a conservative, ischemia-guided strategy in the absence of ongoing symptoms or high-risk features. A consensus emerged around stratifying patients by risk to guide the timing of intervention. Consistent with international guidelines, high-risk patients (e.g., GRACE score >140) should ideally undergo an early invasive strategy within 24 hours. However, panelists acknowledged that, in some regional settings, logistical and resource constraints may necessitate extending this timeframe to 24-72 hours while prioritizing timely access to invasive evaluation. Intermediate-risk patients (e.g., GRACE score <140) warrant an individualized approach based on ischemia assessment, whereas low-risk patients may be managed conservatively. While ESC guidelines remain the foundation for clinical decision-making, panelists emphasized the importance of adapting these recommendations to real-world constraints, particularly in resource-limited settings.

Socioeconomic Considerations and Future Directions

Expert discussion also revealed divergent opinions regarding long-term antiplatelet strategies beyond the first year post-PCI. Although some clinicians supported maintenance with aspirin or ticagrelor monotherapy, others emphasized the need for more personalized decisions based on stent complexity, comorbidities, and overall thrombotic risk. Across all discussions, there was broad agreement that socioeconomic factors, including medication availability, out-of-pocket costs, and procedural accessibility, significantly shape real-world DAPT choices in Lebanon. These practical limitations often necessitate individualized approaches that adapt international guideline recommendations to the realities of daily clinical practice (Figure 1).

## Conclusions

The management of DAPT after PCI has entered a new era of individualization, guided by both rigorous evidence and clinical expertise. Latest guidelines (2022-2025) from ACC/AHA and ESC no longer prescribe a rigid DAPT duration for all, but instead provide a framework to tailor therapy to the patient’s risk profile. Major randomized trials have expanded our understanding: many patients, particularly those at elevated bleeding risk, achieve comparable ischemic outcomes with shorter DAPT durations while reducing bleeding by early discontinuation of one antiplatelet agent. Conversely, patients with a high residual ischemic risk may benefit from prolonged or intensified antithrombotic therapy, including extended DAPT or the addition of low-dose anticoagulation, provided their bleeding risk is low. Selection of the P2Y12 inhibitor should be individualized according to the patient's ischemic and bleeding risks, clinical characteristics, comorbidities, tolerability, and local availability. Prasugrel may be preferred in selected patients undergoing PCI without contraindications, whereas clopidogrel remains appropriate when guided by clinical indications, bleeding risk, or when more potent agents are contraindicated or unavailable. Ticagrelor has the broadest body of evidence supporting both standard and abbreviated DAPT strategies, although its use may be limited by dyspnea, bleeding risk, bradyarrhythmia, tolerability, cost, or availability. In the opinion of the expert panel, the breadth of evidence supporting ticagrelor across different treatment strategies makes it an attractive option for many patients when clinically appropriate.

During recent expert panel discussions in cardiology forums, a common theme has been “personalize, personalize, personalize.” Experts emphasize that decisions on DAPT should incorporate clinical factors (age, comorbidities, prior bleeding), anatomic factors (complexity of PCI, number of stents), and even patient preferences. Risk scores like PRECISE-DAPT and DAPT score are viewed as useful tools to aid (but not dictate) these decisions. There is also recognition that we must be agile in de-intensifying or re-intensifying therapy as a patient’s condition evolves: for instance, if a patient develops a bleeding ulcer at month 3, we should abbreviate DAPT; if a patient tolerates a year of DAPT and has extensive disease, we might extend it. The days of an automatic 12-month DAPT for everyone have given way to a more nuanced approach.

Another point of expert consensus is the importance of patient education and shared decision-making. Since there is often no one “right answer” for all patients, discussing the risks and benefits of various DAPT strategies with the patient is key. For example, some patients may accept a higher bleeding risk to know they are doing everything to prevent a heart attack, while others are very bleed-averse; these preferences can inform whether to continue aggressive therapy or shorten it. Guidelines increasingly acknowledge patient values in prolonged therapy decisions (Class IIb recommendations for extended DAPT often come with the caveat of “if the patient is tolerating and desires continued therapy”).

In conclusion, the current state of DAPT after PCI is one of precision medicine: using the latest evidence, clinicians should customize DAPT duration and intensity for each individual. Ongoing trials are comparing some of these approaches directly (for instance, short DAPT vs. de-escalation head-to-head), which will further inform practice. But already, the trend is clear: we have moved from uniform protocols to an era of DAPT à la carte, guided by clinical judgment, trial evidence, and patient-centered considerations. By adhering to updated guidelines and applying expert insight, cardiologists and interventionists can improve outcomes while minimizing harm, ensuring each patient gets the right DAPT for the right duration, in the context of their unique clinical picture. This expert perspective aligns with the latest recommendations and underscores that managing DAPT is as much an art as a science, one that continues to evolve with each new study and each patient we treat.

## References

[1] Kereiakes DJ (2025). Dual antiplatelet therapy duration following percutaneous coronary intervention: Time for a change. J Soc Cardiovasc Angiogr Interv.

[2] Mendieta G, Mehta S, Baber U (2023). Bleeding and ischemic risks of ticagrelor monotherapy after coronary interventions. J Am Coll Cardiol.

[3] Yang S, Kang J, Park KW (2023). Comparison of antiplatelet monotherapies after percutaneous coronary intervention according to clinical, ischemic, and bleeding risks. J Am Coll Cardiol.

[4] Rao SV, O'Donoghue ML, Ruel M (2025). 2025 ACC/AHA/ACEP/NAEMSP/SCAI guideline for the management of patients with acute coronary syndromes: A report of the American College of Cardiology/American Heart Association Joint Committee on clinical practice guidelines. Circulation.

[5] Manla Y, Almahmeed W (2023). The pandemic of coronary heart disease in the Middle East and North Africa: What clinicians need to know. Curr Atheroscler Rep.

[6] Zeidan RK, Farah R, Chahine MN, Asmar R, Hosseini H, Salameh P, Pathak A (2016). Prevalence and correlates of coronary heart disease: First population-based study in Lebanon. Vasc Health Risk Manag.

[7] Isma'eel HA, Almedawar MM, Breidy J (2018). Worsening of the cardiovascular profile in a developing country: The Greater Beirut Area Cardiovascular Cohort. Glob Heart.

[8] Byrne RA, Rossello X, Coughlan JJ (2023). 2023 ESC guidelines for the management of acute coronary syndromes. Eur Heart J.

[9] Levine GN, Bates ER, Bittl JA (2016). 2016 ACC/AHA guideline focused update on duration of dual antiplatelet therapy in patients with coronary artery disease: A Report of the American College of Cardiology/American Heart Association Task Force on clinical practice guidelines: An update of the 2011 ACCF/AHA/SCAI guideline for percutaneous coronary intervention, 2011 ACCF/AHA guideline for coronary artery bypass graft surgery, 2012 ACC/AHA/ACP/AATS/PCNA/SCAI/STS guideline for the diagnosis and management of patients with stable ischemic heart disease, 2013 ACCF/AHA guideline for the management of ST-elevation myocardial infarction, 2014 AHA/ACC guideline for the management of patients with non-ST-elevation acute coronary syndromes, and 2014 ACC/AHA guideline on perioperative cardiovascular evaluation and management of patients undergoing noncardiac surgery. Circulation.

[10] Valgimigli M, Frigoli E, Heg D (2021). Dual antiplatelet therapy after PCI in patients at high bleeding risk. N Engl J Med.

[11] Mauri L, Kereiakes DJ, Yeh RW (2014). Twelve or 30 months of dual antiplatelet therapy after drug-eluting stents. N Engl J Med.

[12] Bonaca MP, Bhatt DL, Steg PG (2016). Ischaemic risk and efficacy of ticagrelor in relation to time from P2Y12 inhibitor withdrawal in patients with prior myocardial infarction: Insights from PEGASUS-TIMI 54. Eur Heart J.

[13] Steg PG, Bhatt DL, Simon T (2019). Ticagrelor in patients with stable coronary disease and diabetes. N Engl J Med.

[14] Bonaca MP, Bhatt DL, Cohen M (2015). Long-term use of ticagrelor in patients with prior myocardial infarction. N Engl J Med.

[15] Wallentin L, Becker RC, Budaj A (2009). Ticagrelor versus clopidogrel in patients with acute coronary syndromes. N Engl J Med.

[16] Wiviott SD, Braunwald E, McCabe CH (2007). Prasugrel versus clopidogrel in patients with acute coronary syndromes. N Engl J Med.

[17] Sun M, Cui W, Li L (2021). Comparison of clinical outcomes between ticagrelor and clopidogrel in acute coronary syndrome: A comprehensive meta-analysis. Front Cardiovasc Med.

[18] Jiang Z, Liu L, Bundhun PK (2023). Cardiovascular outcomes observed with ticagrelor versus clopidogrel in type 2 diabetes mellitus patients with acute coronary syndrome: A meta-analysis. Diabetes Ther.

[19] Collet JP, Thiele H, Barbato E (2021). 2020 ESC guidelines for the management of acute coronary syndromes in patients presenting without persistent ST-segment elevation. Eur Heart J.

[20] Mehran R, Baber U, Sharma SK (2019). Ticagrelor with or without aspirin in high-risk patients after PCI. N Engl J Med.

[21] Kim BK, Hong SJ, Cho YH (2020). Effect of ticagrelor monotherapy vs ticagrelor with aspirin on major bleeding and cardiovascular events in patients with acute coronary syndrome: The TICO randomized clinical trial. JAMA.

[22] Watanabe H, Morimoto T, Natsuaki M (2022). Comparison of clopidogrel monotherapy after 1 to 2 months of dual antiplatelet therapy with 12 months of dual antiplatelet therapy in patients with acute coronary syndrome: The STOPDAPT-2 ACS randomized clinical trial. JAMA Cardiol.

[23] Cuisset T, Deharo P, Quilici J (2017). Benefit of switching dual antiplatelet therapy after acute coronary syndrome: The TOPIC (timing of platelet inhibition after acute coronary syndrome) randomized study. Eur Heart J.

[24] Kim CJ, Park M-W, Kim MC (2021). Unguided de-escalation from ticagrelor to clopidogrel in stabilised patients with acute myocardial infarction undergoing percutaneous coronary intervention (TALOS-AMI): An investigator-initiated, open-label, multicentre, non-inferiority, randomised trial. Lancet.

[25] Sibbing D, Aradi D, Jacobshagen C (2017). Guided de-escalation of antiplatelet treatment in patients with acute coronary syndrome undergoing percutaneous coronary intervention (TROPICAL-ACS): A randomised, open-label, multicentre trial. Lancet.

[26] Claassens DM, Vos GJ, Bergmeijer TO (2019). A genotype-guided strategy for oral P2Y(12) inhibitors in primary PCI. N Engl J Med.

[27] McClure JD, Ramsay JC, Berry C (2020). Pooled analysis of bleeding, major adverse cardiovascular events, and all-cause mortality in clinical trials of time-constrained dual-antiplatelet therapy after percutaneous coronary intervention. J Am Heart Assoc.

[28] Tavenier AH, Mehran R, Chiarito M (2022). Guided and unguided de-escalation from potent P2Y12 inhibitors among patients with acute coronary syndrome: A meta-analysis. Eur Heart J Cardiovasc Pharmacother.

[29] Ge Z, Kan J, Gao X (2024). Ticagrelor alone versus ticagrelor plus aspirin from month 1 to month 12 after percutaneous coronary intervention in patients with acute coronary syndromes (ULTIMATE-DAPT): A randomised, placebo-controlled, double-blind clinical trial. Lancet.

[30] Hong SJ, Lee SJ, Suh Y (2024). Stopping aspirin within 1 month after stenting for ticagrelor monotherapy in acute coronary syndrome: The T-PASS randomized noninferiority trial. Circulation.

[31] Eikelboom JW, Connolly SJ, Bosch J (2017). Rivaroxaban with or without aspirin in stable cardiovascular disease. N Engl J Med.

[32] Mega JL, Braunwald E, Wiviott SD (2012). Rivaroxaban in patients with a recent acute coronary syndrome. N Engl J Med.

